# High grade serous ovarian carcinomas originate in the fallopian tube

**DOI:** 10.1038/s41467-017-00962-1

**Published:** 2017-10-23

**Authors:** S. Intidhar Labidi-Galy, Eniko Papp, Dorothy Hallberg, Noushin Niknafs, Vilmos Adleff, Michael Noe, Rohit Bhattacharya, Marian Novak, Siân Jones, Jillian Phallen, Carolyn A. Hruban, Michelle S. Hirsch, Douglas I. Lin, Lauren Schwartz, Cecile L. Maire, Jean-Christophe Tille, Michaela Bowden, Ayse Ayhan, Laura D. Wood, Robert B. Scharpf, Robert Kurman, Tian-Li Wang, Ie-Ming Shih, Rachel Karchin, Ronny Drapkin, Victor E. Velculescu

**Affiliations:** 1 Department of Medical Oncology, Dana-Farber Cancer Institute and Harvard Medical School, Boston, MA 02215 USA; 2 Sidney Kimmel Comprehensive Cancer Center, Johns Hopkins University School of Medicine, Baltimore, MD 21287 USA; 3 Department of Biomedical Engineering, Institute for Computational Medicine, Johns Hopkins University, Baltimore, MD 21218 USA; 4 Department of Computer Science, Institute for Computational Medicine, Johns Hopkins University, Baltimore, MD 21218 USA; 5 Personal Genome Diagnostics, Baltimore, MD 21224 USA; 6 Department of Pathology, Brigham and Women’s hospital and Harvard Medical School, Boston, MA 02115 USA; 7 Department of Pathology, University of Pennsylvania Perelman School of Medicine, Philadelphia, PA 19104 USA; 8 Division of Clinical Pathology, Faculty of Medicine, Geneva University Hospital, 1205 Geneva, Switzerland; 9 Department of Pathology, Seirei Mikatahara Hospital, Hamamatsu, 433-8558 Japan; 10 Department of Tumor Pathology, Hamamatsu University School of Medicine, Hamamatsu, 431-3192 Japan; 11 Department of Molecular Pathology, Hiroshima University School of Medicine, Hiroshima, 739-0046 Japan; 12 Departments of Gynecology and Obstetrics and Pathology, Johns Hopkins University School of Medicine, Baltimore, MD 21287 USA; 13 Present Address: Department of Oncology, Geneva University Hospitals, Geneva, 1205 Switzerland; 14 Personal Genome Diagnostics, Baltimore, MD 21224 USA; 15 Present Address: Department of Pathology, Beth Israel Deaconess Medical Center, Boston, MA 02215 USA; 16 Present Address: Department of Obstetrics and Gynecology, Penn Ovarian Cancer Research Center, University of Pennsylvania Perelman School of Medicine, Philadelphia, PA 19104 USA

## Abstract

High-grade serous ovarian carcinoma (HGSOC) is the most frequent type of ovarian cancer and has a poor outcome. It has been proposed that fallopian tube cancers may be precursors of HGSOC but evolutionary evidence for this hypothesis has been limited. Here, we perform whole-exome sequence and copy number analyses of laser capture microdissected fallopian tube lesions (p53 signatures, serous tubal intraepithelial carcinomas (STICs), and fallopian tube carcinomas), ovarian cancers, and metastases from nine patients. The majority of tumor-specific alterations in ovarian cancers were present in STICs, including those affecting *TP53, BRCA1*, *BRCA2* or *PTEN*. Evolutionary analyses reveal that p53 signatures and STICs are precursors of ovarian carcinoma and identify a window of 7 years between development of a STIC and initiation of ovarian carcinoma, with metastases following rapidly thereafter. Our results provide insights into the etiology of ovarian cancer and have implications for prevention, early detection and therapeutic intervention of this disease.

## Abstract (web summary)

It has previously been proposed that high-grade serous ovarian carcinoma (HGSOC) may originate from the fallopian tube. Here, the authors analyze genetic aberrances in fallopian tube lesions, ovarian cancers, and metastases from HGSOC patients and establish the evolutionary origins of HGSOC in the fallopian tube.

## Introduction

Ovarian cancer is the leading cause of death from gynecologic cancers^1, 2^. The 10-year survival is < 30% and has not improved significantly over the last 30 years^3^. Despite significant efforts, various screening and therapeutic strategies have generally not led to improved overall survival^4, 5^. One of the major challenges to improved diagnostic and therapeutic intervention in ovarian cancer has been a limited understanding of the natural history of the disease. Ovarian carcinoma is a highly heterogeneous group of diseases including different histological subtypes with distinct clinicopathological and molecular genetic features that can be generally classified as Type I and Type II tumors^6^. Among them, high-grade serous ovarian carcinoma (HGSOC, the major Type II tumor) is the most common histologic subtype of ovarian cancer, accounting for three quarters of ovarian carcinoma^7–10^. Genomic analyses of HGSOC have identified genetic alterations in *TP53, BRCA1, BRCA2, PTEN*, and other genes although few of these discoveries have affected clinical care^11, 12^. HGSOC is diagnosed at advanced stages in ~70% of cases, and these women have a significantly worse outcome than those with early stage disease. Until recently, the prevailing view of HGSOC was that it developed from the ovarian surface epithelium. However, early in situ lesions that arise from the ovarian surface epithelium and progress to invasive HGSOC have never been reproducibly identified.

Insights into the pathogenesis of HGSOC have emerged from investigating the prevalence of occult ovarian and fallopian tube (FT) carcinomas in women with germline mutations of *BRCA1/BRCA2* genes^13–17^. Potential precursor lesions of HGSOC were identified in the fimbriae of the FTs removed as part of prophylactic surgery^16^. Such lesions, including a *TP53* mutant single-cell epithelial layer (p53 signature) and serous tubal intraepithelial carcinoma (STIC)^17, 18^, have been identified in patients with advanced stage sporadic HGSOC of the ovary, FT and peritoneum^18^. Immunohistochemical as well as targeted sequencing analyses have shown that FT lesions harbor the same *TP53* mutation as surrounding invasive carcinomas^17–21^. These analyses suggest a clonal relationship among such tumors but given the limited number of genes analyzed do not conclusively identify the initiating lesions nor exclude the possibility of FT metastases from primary ovarian carcinomas^21, 22^. Yet additional studies have evaluated clonal intraperitoneal spread of ovarian cancer using whole genome analyses, but these efforts did not analyze precursor lesions such as STICs that may give rise to this disease^23^.

In this study, we use exome-wide sequence and structural analyses of multiple tumor samples from the same individual to examine the origins of HGSOC. We have previously shown that the acquisition of somatic alterations can be used as a molecular marker in the development of human cancer^24^. Here, we examine whether the compendium of somatic alterations identified in different lesions may provide insights into the evolutionary relationship between primary FT lesions, including p53 signatures and STIC lesions, ovarian carcinomas, and intraperitoneal metastases.

## Results

### Overall approach

To elucidate the relationship among tumors in patients with HGSOC, we performed whole-exome sequencing of 37 samples from five patients diagnosed with sporadic HGSOC who underwent upfront debulking (Supplementary Data 1). This included STIC lesions, FT carcinomas, and ovarian cancers in all five patients; appendiceal, omental, or rectal metastases in three of patients (CGOV62, CGOV280, CGOV278); p53 signatures in two patients (CGOV62, CGOV63); and a STIC lesion in the contralateral FT from the affected ovarian cancer (CGOV280).

**Fig. 1:**
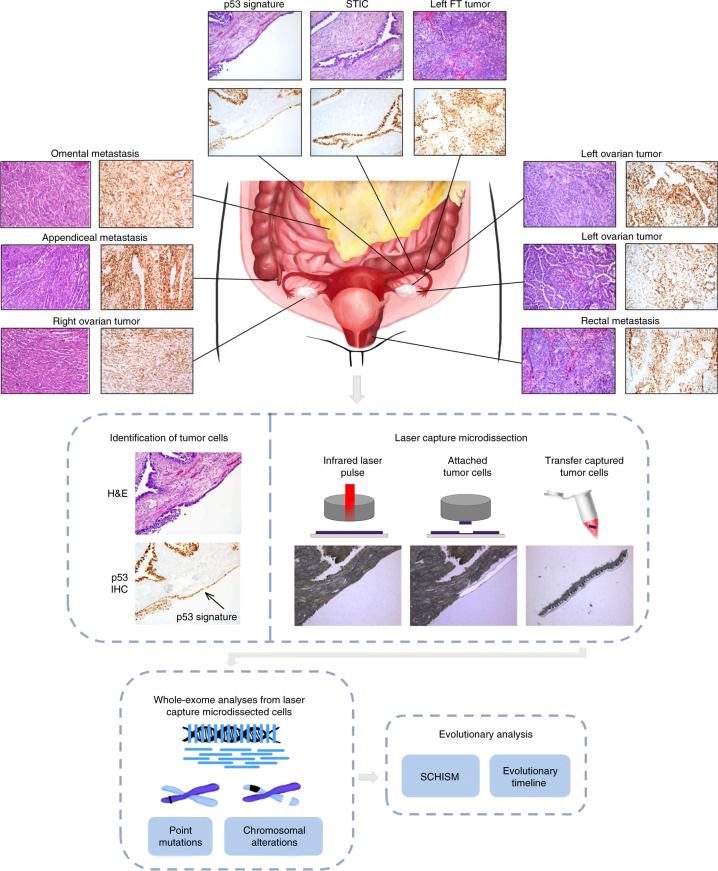
Schematic of sample isolation and next-generation sequencing analyses. (Top panel) Tumor sites analyzed from CGOV62 with stage III HGSOC. For each sample, slides were stained with hematoxylin and eosin as well as analyzed by immunohistochemical staining of p53. (Middle panel) Tumor samples were microdissected for genomic analyses. For microdissection for STIC and p53 signature lesions, tumor cells were identified using immunohistochemical staining of p53 and isolated through laser capture microdissection. (Bottom panel, left) Next-generation sequencing analyses were performed for tumor specimens using either whole-exome or targeted analyses. (Bottom panel, right) Somatic mutations and chromosomal alterations were used to evaluate tumor evolution using the tumor subclonality phylogenetic reconstruction algorithm SCHISM and to determine a timeline for tumor progression

In addition, we analyzed isolated STIC lesions from four patients (CGOV64, CGOV65, CGOV303, and CGOV304), three of whom had germline pathogenic *BRCA* alterations and underwent prophylactic bilateral salpingo-oophorectomy, and a fourth who had bilateral salpingo-oophorectomy and hysterectomy in the context of a pelvic mass (Supplementary Data 1). For all patients, laser capture microdissection (LCM) was used to isolate lesions after immunohistochemistry (IHC) staining of p53 in STICs and p53 signatures if these contained a *TP53* missense mutation or after hematoxylin staining if the samples contained a *TP53* nonsense mutation (Fig. 1). All other samples were microdissected after hematoxylin staining. Whole blood, normal ovarian stroma, normal FT stroma, or normal cervix were used as control samples.

To identify genetic alterations in the coding regions of these cancers, we used next-generation sequencing platforms to examine entire exomes in matched tumor and normal specimens of all patients (Fig. 1). This approach allowed us to identify non-synonymous and synonymous sequence changes, including single base and small insertion or deletion mutations, as well as copy number alterations in coding genes. Given the challenges of exome-wide analyses of small tumor samples observed in STICs and p53 signature lesions, we developed experimental and bioinformatic approaches for detection of somatic alterations from laser capture microdissected tissue. These included optimized approaches for microdissection of STICs and p53 signatures after immunohistochemical staining, improved DNA recovery from laser captured material, library construction from limited and stained tissue samples, and error correction methods in next-generation sequence analyses (Methods section). The analyses of p53 signatures were particularly challenging because these are extremely small lesions, representing 10–30 cells per section and less than several hundred cells total that result in minute amounts (less than a few ng) of isolated DNA. We optimized these approaches using a targeted next-generation sequencing approach analyzing 120 genes in a subset of samples from patient CGOV62, and then used whole-exome analyses to evaluate coding sequence alterations in all samples (Supplementary Data 2–4). We obtained a total of 719 Gb of sequence data, resulting in an average per-base sequence ~178-fold total coverage (~112-fold distinct coverage) for each tumor analyzed by whole-exome sequencing (Supplementary Data 2).

### Analysis of sequence and structural changes

**Fig. 2:**
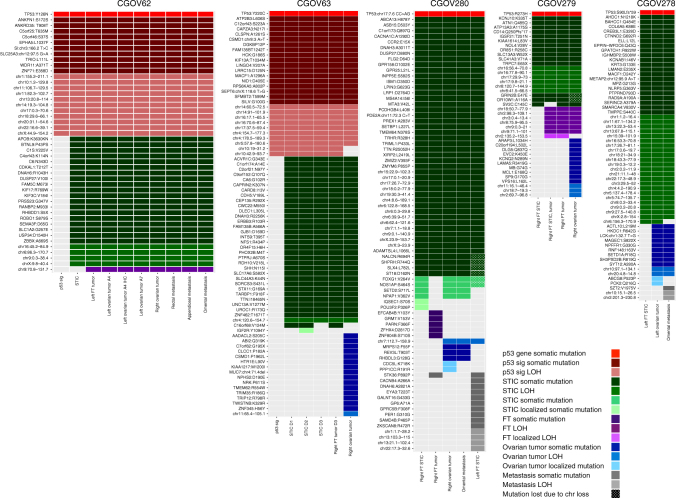
Somatic mutation and allelic imbalance profiles among different tumor lesions. Somatic mutations and segments of allelic imbalance detected by whole-exome analyses are indicated as colored cells in rows for all patients. Darker shades of each color indicate somatic mutations while lighter shades indicate allelic imbalances. The tumor samples analyzed for each patient are indicated in columns (p53 sig, p53 signature; STIC, serous tubal intraepithelial carcinoma). For ovarian tumors in CGOV62 and STIC lesions in CGOV63 multiple blocks are indicated, including one ovarian tumor where multiple sections were analyzed after hematoxylin and eosin staining or after immunohistochemistry (IHC) staining of p53. These analyses indicated that staining methods did not affect detection of somatic alterations. The color of mutations indicates the degree of relatedness among tumor samples: red, shared among all tumor samples with TP53 highlighted at the top row; green, shared among all tumor samples except p53 signature lesion; purple, shared among fallopian tube tumor and omental metastasis; blue indicates mutations that were first detected in the ovarian tumors; and gray indicates mutations that were only detected in metastatic lesions. Additional color shades or patterns indicate mutations that are localized to specific lesions or lost due to chromosome loss as shown in the legend

Whole-exome sequence analyses of the tumor samples from each patient identified somatic mutations that were present in all neoplastic samples analyzed as well as specific changes that were present in individual or subsets of tumors (Fig. 2). As expected, we identified sequence changes in the *TP53* tumor suppressor gene, a well-known driver gene in HGSOC, in all cases. The *TP53* alterations were identical in all samples analyzed for each patient including in the p53 signatures, the STIC lesions, and other carcinomas. These data suggest that mutation of *TP53* was among the earliest initiating events for HGSOC development as all lesions harbored this alteration.

IHC staining for p53 did not identify any nuclear positive staining of p53 on the ovarian surface epithelium in any of the cases that had *TP53* missense mutation, whereas all carcinomas, STICs, and p53 signatures in the FT were positive. Whole-exome sequence analyses of normal ovarian stroma (no p53 staining) microdissected from three patients (CGOV64, CGOV65, CGOV280) did not find any genomic abnormalities. Analysis of the resected tissues revealed that none of the nine cases had ovarian inclusion cysts. These observations suggest that there is no early lesion with *TP53* mutation in the surface epithelium or other normal regions within the ovary.

Because *TP53* mutations are expected to be clonal and were all homozygous due to loss of heterozygosity (LOH) of the remaining wild-type allele (as determined in our subsequent allelic imbalance analyses), we used the mutant allele fraction of *TP53* in each sample to estimate tumor purity. We further analyzed sequence alterations in all samples with estimated tumor purities > 50%, while four samples with tumor cellularities below this threshold (omental metastasis from CGOV279 and right ovarian tumor from CGOV278) or that were miliary carcinomas (rectal and sigmoidal metastases from CGOV63) were only analyzed for structural changes.

Using a high-sensitivity mutation detection pipeline, we identified an average of 33 non-synonymous and synonymous sequence alterations per tumor sample. Candidate alterations were evaluated across samples in an individual to determine if they were present in multiple neoplastic lesions or were unique to a particular sample. To allow for the possibility that a subclone may have developed in a tumor lesion prior to becoming a dominant clone at another location, we determined if genetic alterations that were present in one tumor were also present in a low fraction of neoplastic cells of other lesions. This method required high coverage of analyzed alterations in all samples and excluded potential artifacts related to mapping, sequencing or PCR errors, allowing specific detection of alterations present in ≥ 1% of sequence reads (see Methods section for additional information).

The composition of sequence alterations was relatively similar among the affected lesions of each patient. For example, for CGOV62, the STIC lesion, FT carcinomas, left and right ovarian cancers, and all three metastatic lesions harbored a common set of somatic mutations (Fig. 2). In CGOV63, CGOV279, and CGOV278, while most of the sequence alterations were the same among the tumors of each patient, a subset of mutations could distinguish the STIC lesions and FT carcinomas from ovarian cancers or intraperitoneal metastases.

**Fig. 3:**
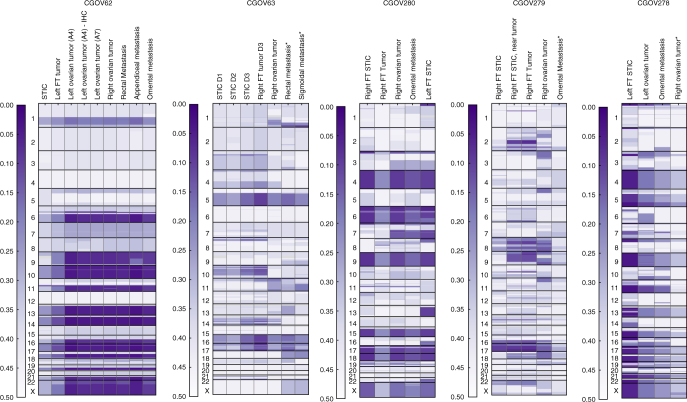
Genome-wide allelic imbalance profile. Minor allele frequency of heterozygous SNPs identified from normal tissue in each patient are derived in each tumor sample, enabling assessment of allelic imbalance in ~17,000 loci across the exome. Circular binary segmentation (CBS) is applied to minor allele frequencies of SNPs with minimum coverage of 10× in each tumor sample, and the resulting segment means are shown as a heatmap. Asterisks indicate samples where corresponding mutation analyses were not performed due to low tumor purity (omental metastasis of CGOV279, right ovarian tumor of CGOV278) or miliary pattern of tumor samples (peritoneal metastases of CGOV63). Given the relatively lower number of distinct DNA molecules available from the p53 signature samples from CGOV62 and CGOV63, these samples were subjected to a more sensitive LOH analysis (Methods, Genome-wide imbalance analysis) and are not shown here

Given the importance of chromosomal instability in HGSOC^11^, we extended our analyses to examine structural variation in the multiple tumors of each patient. We focused on regions of allelic imbalance that can result from the complete loss of an allele (LOH) or from an increase in copy number of one allele relative to the other. We divided the genome into chromosome segments and for each segment compared the minor allele (B-allele) frequency values in tumor and normal samples using the ~17,000 whole-exome germline heterozygous single-nucleotide polymorphisms (SNPs) observed (Fig. 3, Supplementary Figs. 1–9 and Supplementary Data 7–11). Overall, we observed that an average of ~26% (range 12–39%) of the genome had chromosomal imbalances in the samples analyzed (Fig. 3).

Integration of sequence and structural alterations identified an average of 47 alterations per sample (range 21–74) (Fig. 2). The combination of both types of alterations allowed robust genomic differentiation between STICs and ovarian cancers or metastatic lesions in all patients analyzed. In patient CGOV62, a LOH of 9q (70.8–131.7 Mb) provided a clear difference between the STIC and all other carcinomas analyzed (Figs. 2 and 3). Likewise, chromosomal changes in 7q represented a distinguishing feature between the right STIC or right FT tumors and the remaining lesions (ovarian cancers, omental metastasis, and left STIC) in CGOV280 (Figs. 2 and 3). In patient CGOV279, multiple regions of allelic imbalance were present in a STIC near the FT carcinoma, while these were absent in a STIC that was not adjacent to this lesion.

### Evolutionary relationship of neoplastic lesions

**Fig. 4:**
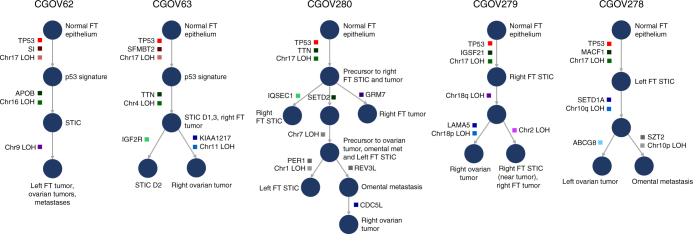
Schematic of tumor evolution. The history of tumor evolution in each patient is modeled as a subclonal hierarchy inferred from the somatic mutations and large scale genomic regions harboring loss of heterozygosity (LOH features) using the SCHISM framework, and is depicted as a tree. Each tree starts from a root node corresponding to the normal fallopian tube epithelium (germline). In all patients, mutations in *TP53* (red boxes) are among the earliest somatic alterations and are ubiquitously present in all tumor samples. Somatic alterations (boxes) are acquired along edges (arrows) of the tree, and example alterations are indicated in each case. Nodes of the tree represent cells whose genotype is described by the presence of somatic mutations and LOH features on the path connecting the node to the root of the tree. Each node is labeled with tumor samples harboring all upstream and lacking any downstream alterations. The trees inferred for all patients support a pattern of evolution with p53 signatures and STIC lesions as early events in tumorigenesis. Mutation clusters and LOH feature groups follow the same color code as Fig. 2

As somatic genetic alterations can be used to recreate the evolutionary history of tumor clones, we used the somatic sequence mutations and chromosomal alterations observed in each patient to determine the history of tumor clonal evolution. We employed a subclone hierarchy inference tool called SCHISM (SubClonal Hierarchy Inference from Somatic Mutations) which enables improved phylogenetic reconstruction by incorporating estimates of the fraction of neoplastic cells in which a mutation occurs (mutation cellularity)^25^. We estimated the cellularity of each mutation by correcting the observed allele frequencies for tumor purity and copy number levels (Methods section). In addition to the observed structural alterations, this approach allowed us to use 213 synonymous and non-synonymous somatic sequence alterations to construct the phylogenetic trees illustrated in Fig. 4 and Supplementary Data 5.

A SCHISM tree node represents cells harboring a unique compartment of mutations defining a subclone whereas an edge represents a set of mutations acquired by the cells in the progeny nodes that distinguish them from the cells in the parental node. By definition, for an individual cancer there could only be one parental clone, although there could be many different progeny subclones representing invasive or metastatic lesions or further evolution of the primary tumor. The optimal hierarchy among subclones is determined by examining all possible pairwise relationships between somatic alterations, and performing a heuristic search over the space of phylogenetic trees to identify a model that best explains the observed alterations.

In all samples, the SCHISM analysis of sequence and structural alterations suggested that the p53 signature or STIC lesions contained the ancestral clone for the observed cancers (Fig. 4). This evolutionary relationship was strengthened by the observation that nearly all of the alterations within the p53 signature and STIC lesions were shared by all other lesions. For example, the ovarian tumors of all cases displayed alterations that were shared in FT lesions but also contained additional changes, suggesting that these represented daughter clones of the latter tumors (Fig. 2). Likewise, the ovarian cancers or their immediate precursors were likely the direct parental clones for the metastases in CGOV62, CGOV278, and CGOV280 as demonstrated by the shared alterations that were not contained in earlier FT lesions. Overall, the phylogenetic model generated by these data suggests a progression from FT epithelium to p53 signatures and to STIC lesions which are then precursors of FT carcinoma, ovarian carcinoma, and metastatic lesions. In addition to the sequential accumulation of alterations in this linear evolution, we also observed branching phylogenetic trees due to continued evolution within STIC lesions as well as FT carcinomas and ovarian carcinomas (Fig. 4). We compared evolutionary trees resulting from SCHISM analysis with those derived by maximum parsimony phylogeny using PHYLIP and the results were similar in all cases (Fig. 4 and Supplementary Fig. 11).

Interestingly, patient CGOV280 had a right STIC, a right fallopian carcinoma, and a right ovarian cancer but also had a STIC in the left FT (Supplementary Fig. 5). In this case the SCHISM analysis suggested that the lesion in the left FT which was pathologically determined to be a STIC actually represented a metastatic lesion of the right ovarian cancer (Fig. 4). This lesion shared nearly all the alterations of the ovarian cancer but contained 10 single base substitutions and four additional regions of allelic imbalance on chromosomes 1, 13, and 22, and both the left STIC and right ovarian cancer had an additional region of allelic imbalance on chromosome 7 that was absent in the right STIC (Figs. 2 and 3). These observations are consistent with the above model of STIC to ovarian cancer progression, but suggest that in advanced disease ovarian cancers may also seed metastatic deposits throughout the peritoneum, including to the FT on the contralateral side.

### Genomic alterations in isolated STICs

Neoplastic cells observed in the FTs rather than the ovaries removed from carriers of germline mutation of *BRCA1* and *BRCA2* provided the first indication of the FT as a potential cell of origin of HGSOC^15, 26^. Since < 1.25% of HGSOC are diagnosed with stage I disease^22^, *BRCA* carriers provide a unique opportunity to analyze genomic alterations in isolated STICs without associated HGSOC. We examined neoplastic samples from three individuals with germline *BRCA* alterations where STIC lesions were incidentally identified after prophylactic bilateral salpingo-oophorectomy, and one patient where two STICs were identified after resection of a pelvic mass (Supplementary Data 1). We identified *BRCA1 or BRCA2* sequence alterations or deletions in the germline of three of these patients (*BRCA1* Q1200X, *BRCA2* L2653P, and a *BRCA2* 55 kb hemizygous deletion in CGOV65, CGOV64, and CGOV304, respectively), as well as somatic mutations in *TP53*, and LOH of both chromosome 13 and 17, encompassing the *BRCA1, BRCA2*, and *TP53* loci in all of these cases (Supplementary Figs. 6, 7, 8, and 9). Whole-exome analyses showed that the STIC lesions contained a total of 91, 23, 34, and 46 non-synonymous and synonymous somatic mutations, in CGOV65, CGOV64, CGOV303, and CGOV304, respectively. Overall, these analyses revealed that STICs in isolation in patients with or without germline *BRCA* changes have a roughly similar number of sequence changes to STICs in patients with sporadic tumors. These observations provide evidence that isolated STICs may act as precursors in the same manner as those identified in patients with sporadic advanced stages HGSOC analyzed in this study.

### Recurrent molecular alterations

We examined tumors from the nine patients to identify recurrent non-silent sequence or chromosomal changes. Although no genes other than *TP53* were mutated in all patients analyzed, we identified mutations in ten genes that were altered in two or more patients (Supplementary Data 6). These included mutations in the tumors of two patients of the *PIK3R5* gene that encodes a regulatory subunit of the PI3-kinase complex. CGOV64 also had a somatic alteration in *PTEN* that together with changes in *PIK3R5* highlight the importance of the PI3K pathway in ovarian cancer^11^. Additional genes that were observed to be altered in other ovarian cancers through other large scale sequencing efforts such as TCGA^11^ are indicated in Supplementary Data 6.

In addition to recurrent sequence changes, we found alterations in regions of allelic imbalances encompassing several tumor suppressor genes involved in ovarian cancer. Remarkably, these included losses of *BRCA1, BRCA2*, and *TP53* in all nine patients, and loss of *PTEN* for CGOV62, CGOV63, CGOV280, and CGOV64 (in addition to the somatic sequence alterations of these genes) (Supplementary Figs. 1–9). In all cases, the LOH observed in the metastatic lesions and ovarian tumor lesions for regions encompassing these genes were already present in the FT tumor and STIC lesions. Considering the evolutionary model above, these data suggest that a combination of sequence changes in a few genes including *TP53* together with loss of the *TP53* wild-type allele as well as *BRCA1, BRCA2*, and *PTEN* may be crucial early events that are needed for the initiation of STICs^27, 28^.

### Evolutionary timeline of ovarian cancer development

To estimate the time between the development of the earliest neoplastic clones in the FT and the development of ovarian and other metastatic lesions we used a mathematical model for comparative lesion analysis^24, 29^. This model estimates the time interval between a founder cell of a tumor of interest and the ancestral precursor cell assuming that mutation rates and cell division times are constant throughout a patient’s life (Methods section). In patient CGOV62, this model would suggest ~1.9 years between the development of the STIC lesion and the ovarian cancer (90% CI, 0.5–4.2 years). For other patients this transition appears to have been slower as the average time between STICs and ovarian cancer among all patients was 6.5 years (1.4–10.7 years). Importantly, in patients with metastatic lesions, the time between the initiation of the ovarian carcinoma and development of metastases appears to have been rapid (average 2 years). There were either no additional mutations in metastatic lesions (e.g., CGOV62 omental, rectal or appendiceal metastasis or CGOV280 omental metastasis) or the number of additional changes was small (e.g., three changes in CGOV278 omental metastasis), reflecting the ease with which cancer cells located on the ovaries can subsequently seed additional peritoneal sites. Although the precise timing of this progression depends on assumptions related to mutation rates, which may change during tumor progression, models employing different rates all showed longer timeline from STIC lesions to ovarian tumors followed by rapid development of metastatic lesions (Methods section).

## Discussion

These results provide a comprehensive evolutionary analysis of sporadic HGSOC in five patients. Given the unique nature of the multiple samples we examined from each patient, our study may have certain limitations not typical of genome-wide efforts. First, the small size of the tumor samples compared to surrounding non-neoplastic tissue could potentially lead to low tumor purity. The high mutant allele fraction of *TP53* among cancer samples (average of 56–85%) indicates that this issue was largely overcome through LCM. Second, the small number of cells in p53 signature samples may have limited our genomic analyses for these lesions. The observation that all sequence changes in p53 signatures were also present in STIC and other carcinomas of multiple sites is consistent with our evolutionary model and suggests that these cells are likely to represent a parental clone of other neoplastic lesions. Third, our analysis was limited to ovarian cancers where STICs and other concomitant lesions were identified, and may therefore not be representative of all HGOCs. The absence of STIC lesions in ~40% of sporadic HGSOCs is likely due to an incomplete sampling of the FT or the overgrowth of the STIC by the carcinoma in the context of bulky disease, but may also reflect another site of origin that has yet to be determined for these cancers^30^. Fourth, this study did not intend to address the intra-tumoral heterogeneity within the carcinomas but rather focused on clonal changes within each tumor. Fifth, as in any evolutionary analyses, the genomic alterations we observed provide the most likely model of tumor development but do not exclude the possibility of other relationships. Nevertheless, our analyses of somatic alterations suggest that models where the ovarian cancer or metastatic lesions seed the FT tumors^20, 21^ (including STICs or p53 signatures) are infrequent and unlikely to be the source of most FT lesions.

Despite these potential limitations, the data we have obtained provide new insights into the etiology of ovarian Type II carcinoma and have significant implications for the prevention, early detection and therapeutic intervention of this disease. The results suggest that ovarian cancer is a disease of the FTs, with the development of p53 signatures and STICs as early events. The subsequent formation of a cancer in the ovaries represents a seeding event from a primary tumor in the FT that already contains sequence and structural alterations in key driver genes, including those in *TP53*, PI3K pathway, and *BRCA1/BRCA2* genes. The recurrent allelic imbalances observed in chromosomes 1, 6, 16, 18, 20, and 22 may suggest additional genes that are involved in this process. The timing of the progression from STICs to ovarian cancer in the cases we analyzed was on average 6.5 years, but seeding of metastatic lesions in these patients occurred rapidly thereafter. This timing is consistent with recent reports showing a difference of 7.7 years in the age of *BRCA* carriers with localized vs. advanced adnexal lesions^31^. This evolutionary timeline can help explain why most HGSOC patients are diagnosed at advanced stage (III/IV) with pelvic and peritoneal spread of disease, and why among asymptomatic *BRCA* germline mutation carriers half of the cases diagnosed with asymptomatic adnexal neoplasia have already seeded to pelvis or peritoneum (> IA)^31^. These observations are largely similar to other genomic analyses of the evolution of ovarian cancer^19, 20, 23, 32^ as well as the recent analyses of STIC lesions that were reported while this study was under review^33^. Our study highlights the role of p53 signatures as early lesions in this evolutionary paradigm.

Our genomic analyses are consistent with population-based studies of the effects of salpingectomy on the risk of ovarian cancer. Prophylactic bilateral salpingo-oophorectomy has been shown to reduce the risk of developing ovarian cancer in *BRCA* mutation carriers to below 5%^34, 35^. Likewise, bilateral salpingectomy, performed as a contraceptive method instead of tubal sterilization, reduced the risk of ovarian cancer by 61% at 10 years^36^. Our study provides a mechanistic basis for these observations and has implications for clinical management in prevention of ovarian cancer. In high risk *BRCA* carriers, bilateral salpingectomy with delayed oophorectomy should be considered^37^ through participation in ongoing clinical trials (NCT02321228; NCT01907789). In non-carriers, our work implies that for women who undergo surgery for benign uterine causes, total abdominal hysterectomy and bilateral salpingectomy with sparing of the ovaries should be considered^38^, and that bilateral salpingectomy may be a preferred contraceptive alternative to tubal ligation. The dual concepts in these recommendations for *BRCA* carriers and non-carriers are that removal of the FTs (rather than the ovaries) may be curative as it eliminates the underlying cellular precursors of ovarian cancer, and that preservation of the ovaries provides long term benefits due to decreased risk and fatalities from coronary heart disease and other illnesses^39^. A limitation of this approach is that as the precise timing of when potentially malignant cells shed from the FT and microscopically seed the ovary is unknown, removal of the tubes may not provide optimal risk-reduction.

Our observations also have implications for improved detection of ovarian cancer. Unfortunately, < 1.25% of HGSOC are confined to the ovary at diagnosis^22^. Earlier detection of this disease is likely to benefit from the identification of a precursor lesion, as has been the case for many other tumor types. Our data suggest that FT neoplasia is the origin of ovarian serous carcinogenesis, and can directly lead to cancer of the ovaries and of other sites. Currently, the typical histopathologic evaluation of FTs typically involves a cursory evaluation of one or two representative sections. Our study suggests that systematic sectioning and extensive examination of total FTs^16^ should become common practice in pathology, and not confined to academic tertiary care centers. Depending on whether the FTs are removed for benign conditions, risk-reducing bilateral salpingectomy, or gynecological cancers, specific examination protocols should be applied^16, 40^. Given the window of time that appears to exist between the formation of FT lesions and development of ovarian cancer, these insights open the prospect of novel approaches for screening. Such approaches may be especially important given the limited therapeutic options currently available for ovarian cancer^4, 5^. Recent advances for ultrasensitive detection of genetic alterations in blood-based liquid biopsies, pap smears, and other bodily fluids^41, 42^, or imaging approaches may provide opportunities in early diagnosis and intervention.

## Methods

### Specimens obtained for sequencing analysis

The study was approved by the Institutional Review Board at Brigham and Women’s Hospital and the Johns Hopkins Hospital and all patients gave informed consent before inclusion. Five sequential patients with stage III sporadic HGSOC, in whom a STIC was identified in their FTs (FT), were included. In addition, we included isolated STICs from three patients with germline *BRCA* deleterious alterations who underwent prophylactic bilateral salpingo-oophorectomy as well as a fourth patient who had bilateral salpingo-oophorectomy and hysterectomy in the context of a pelvic mass. All cases underwent complete tubal examination using the SEE-FIM protocol^16^. Formalin-fixed paraffin embedded (FFPE) blocks were retrieved from the pathology files at Brigham and Women’s Hospital and Johns Hopkins Hospital within the 3 months following surgical diagnosis and stored at 4 °C to slow down nucleic acids degradation. All the cases were reviewed by a gynecologic pathologist (M.S.H., D.I.L., L.S.) that confirmed the diagnosis of STIC and/or p53 signature in the FT. Slides from each FFPE block, including early lesions, invasisve carcinomas and metastases, were stained with hematoxylin and eosin, and analyzed by p53 IHC staining. In each FT, at least one STIC and/or p53 signature was identified and microdissected separately. Importantly, STICs were not pooled together if they were in the same section and were considered separate STICs.

### Immunohistochemistry and laser capture microdissection

For accurate microdissection of early lesions including STIC and p53 signature, IHC staining of p53 was specifically adapted for LCM as previously described^43^. PEN membrane frame slides Arcturus (Life technologies, Carlsbad, CA) were used. Each slide was coated with 350 ul of undiluted poly-l-lysine 0.1% w/v (Sigma, St. Louis, MO). For drying, the slides were placed in a slide holder for 60 min at room temperature. Tissue sections were cut and mounted on the pretreated membrane slides. Deparaffinization was performed in fresh xylene for 5 min twice, followed by 100% ethanol for 2 min, 95% for ethanol 2 min, and 70% ethanol for 2 min. Subsequently, the slides were transferred into distilled water for 5 min. Heat-epitope antigen retrieval (AR) was performed in Citrate Buffer (Dako, Carpinteria, CA) at low temperature (60 °C) for 44 h instead of 120 °C for 10 min to reduce tissue and DNA damage by high temperature. Retrieval solution was pre-warmed to 60 °C before usage. After incubation in the oven, the AR solution was left to cool down to room temperature and the slides were rinsed for 30 seconds in fresh 1×PBS then incubated for 40 min with primary antibody anti-p53 (Epitomics, Burlingame) at 1:100 in a humidifying chamber. Before adding the secondary antibody, slides were washed twice for 1 min in fresh 1×PBS. The secondary antibody, labeled polymer-HRP anti-mouse (Dako EnVision System-HRP (DAB), Carpinteria, CA) was applied for 30 min. Then, slides were washed twice for 1 min in fresh 1×PBS. Chromogenic labeling was performed with 3,3-DAB substrate buffer and DAB chromogen (Dako EnVision System-HRP (DAB), Carpinteria, CA) for 5 min. Slides were washed again for 30 s in fresh distilled water. Dehydration was performed as follows: 70% ethanol for 30 s, 95% ethanol for 30 s, 100% ethanol for 30 s, and xylene for 30 s. The stained slides were microdissected within 2 h with the Arcturus XT LCM system (Life technologies, Carlsbad, CA).

### Hematoxylin staining for laser capture microdissection

Invasive carcinomas from the ovaries, the FTs and intraperitoneal metastases or STICs from patients with negative p53 IHC staining were microdissected after Hematoxylin staining. Briefly, deparaffinization was performed in fresh xylene for 1 min twice followed by 100% ethanol for 1 min, 95% for ethanol 1 min, and 70% ethanol for 1 min. The slides were transferred into distilled water for 2 min before staining with Hematoxylin for 2 min. Subsequently, slides were rinsed in distilled water until they became clear before undergoing dehydration in 70% ethanol for 1 min, 95% ethanol for 1 min, 100% ethanol for 1 min, and xylene for 1 min. The stained slides were microdissected within 2 h.

### Sample preparation and next-generation sequencing

DNA was extracted from patient whole blood using a QIAamp DNA Blood Mini QIAcube Kit (Qiagen Valencia, CA). Genomic DNA from FFPE blocks was extracted from the microdissected tissues using the QIAamp DNA FFPE Tissue kit (Qiagen, Valencia, CA). In brief, the samples were incubated in proteinase K for 16 h before DNA extraction. The digested mixture was transferred to a microtube for DNA fragmentation using the truXTRAC™ FFPE DNA Kit with 10 min shearing time as per the manufacturer’s instructions (Covaris, Woburn, MA). Following fragmentation, the sample was further digested for 24 h followed by 1 h incubation at 80 °C. DNA purification was performed using the QIAamp DNA FFPE Tissue kit following the manufacturer’s instructions (Qiagen, Valencia, CA). Fragmented genomic DNA from tumor and normal samples were used for Illumina TruSeq library construction (Illumina, San Diego, CA) according to the manufacturer’s instructions or as previously described^44^. Exonic or targeted regions were captured in solution using the Agilent SureSelect v.4 kit or a custom targeted panel according to the manufacturer’s instructions (Agilent, Santa Clara, CA). Paired-end sequencing, resulting in 100 bases from each end of the fragments for exome libraries and 150 bases from each end of the fragment for targeted libraries, was performed using Illumina HiSeq 2000/2500 and Illumina MiSeq instrumentation (Illumina, San Diego, CA).

### Next-generation sequencing data and identification of somatic mutations

Somatic mutations were identified using VariantDx^45^ custom software for identifying mutations in matched tumor and normal samples. Prior to mutation calling, primary processing of sequence data for both tumor and normal samples were performed using Illumina CASAVA software (v1.8), including masking of adapter sequences. Sequence reads were aligned against the human reference genome (version hg18 or hg19) using ELAND. Candidate somatic mutations, consisting of point mutations, insertions, and deletions were then identified using VariantDx across either the whole exome or regions of interest^44^. For samples analyzed using targeted sequencing, we identified candidate mutations that were altered in > 10% of distinct reads. For samples analyzed using whole-exome sequencing, we identified candidate mutations that were altered in > 10% of distinct reads with ≥ 5 altered reads in at least one sample, where coverage at the altered base was at least as high as the *TP53* alteration in that sample, and where the ratio of the coverage of the mutated base to the overall sequence coverage of that sample was > 20%. Identified mutations were reported as present in other samples of the same patient if the mutation was present in at least two distinct altered reads. Mutations present in polyN tract ≥ 5 bases, or those with an average distinct coverage below 50× were removed from the analysis.

An analysis of each candidate mutated region was performed using BLAT. For each mutation, 101 bases including 50 bases 5ʹ and 3ʹ flanking the mutated base was used as query sequence (http://genome.ucsc.edu/cgi-bin/hgBlat). Candidate mutations were removed from further analysis, if the analyzed region resulted in > 1 BLAT hits with 90% identity over 70 SCORE sequence length. All candidate alterations were verified by visual inspection.

### Genome-wide allelic imbalance analysis

We performed comparative analysis of LOH across the tumor samples from each patient to identify copy number alterations occurring in the course of tumor evolution. Minor allele frequency (MAF) of germline heterozygous SNPs with minimum coverage of 10× in each tumor sample were segmented using circular binary segmentation algorithm (CBS)^46^. Genomic segments where the difference between tumor and normal MAF exceeded a threshold of 0.10 were labeled as harboring LOH. In each tumor sample, the minimum MAF across segments with minimum size of 10 Mb was calculated to provide a measure of sample purity. Each segment marked as LOH was assigned to one of the three confidence categories: (1) high confidence, segment MAF within 0.1 of the minimum sample MAF. (2) Intermediate confidence, segment MAF within 0.1–0.2 of the minimum sample MAF. (3) Low confidence, segment MAF exceeding the minimum sample MAF by > 0.2.

Next, sample level segments were intersected across the entire set of samples from each patient to derive patient level segments while accounting for the possibility of variable segment break points in different samples (Supplementary Data 7–11). Patient level segments were filtered to keep those covering a minimum of 20 SNPs and with minimum length of 10 Mb. The resulting segments were further narrowed down to only include those with high confidence LOH in at least one of the samples. Genomic segments with LOH in a subset of samples can serve as informative markers to track tumor evolution similar to somatic mutations. To increase the specificity in identifying this class of genomic segments, we required a minimum distance of 0.1 between the MAF of samples with and without LOH. To minimize the possibility of over-segmentation which could result in inflated estimates of the number independent structural alterations, we evaluated patient level segments with boundaries within a 5 Mb window. In cases where the LOH calls were identical and the difference of segment MAFs were ≤0.05 in all tumor samples, the segments were merged.

For CGOV62 and CGOV63, the number of germline heterozygous SNPs meeting the coverage criteria in p53 signature samples was significantly lower than the other samples from the same patient. Thus, we modified the approach above in these two patients to enable sensitive analysis of LOH in p53 signature samples. Initially, the patient level genomic segments of interest were defined excluding p53 signature samples. Next, in each genomic segment, the minor allele of each overlapping germline SNP was determined by taking a majority vote over their minor alleles in the other samples. The coverage and minor allele read count for each SNP was derived using samtools (v0.1.19) mpileup module^47^. The segment MAF in p53 signature samples were calculated by dividing the sum of minor allele read counts across all SNPs by the total coverage of SNPs, circumventing the variance resulting from low coverage at individual SNPs. In each p53 signature sample, segments with MAF lower than that of the normal by at least 0.1 were marked as LOH.

### Copy number analysis

The genome-wide copy number profiles were determined by analysis of the ratio of read counts in the tumor and matched normal whole-exome sequenced samples. In each sample, the number of reads mapping to genomic bins located in target and off-target regions were corrected for biases arising from GC-content, repetitive sequences, and target capture process using CNVKit (v.0.7.6) (https://doi.org/10.1371/journal.pcbi.1004873). The log ratio of the processed tumor to normal read counts provides a measure of copy number in each bin, and was segmented to yield genomic intervals at constant copy number levels. The difference in sequencing library size between the tumor and normal samples is another factor that needs to be accounted for when analyzing reads ratios in NGS-based copy number pipelines. In CNVKit, the log ratio values in each sample are adjusted by setting the median of autosomal bins to 0 in log space, assuming a median ploidy of 2 for the genome. Given the high prevalence of copy number aberrations in ovarian cancer and the high frequency of allelic imbalance in the present cohort, this assumption may not be accurate, and will manifest itself as a genome-wide bias or shift of log ratio values.

Therefore, an alternative approach for normalization of log ratio values was adopted, which takes into account the level of allelic imbalance in each genomic region. Briefly, genomic regions with the least degree of allelic imbalance were identified in each tumor sample, and used in a normalization process based on the notion that these regions can only be present in an even number of copies. The distribution of log ratio values among these regions was inspected to ensure that they belong to the same copy number level. Otherwise, a subset of regions at a common log ratio (and thus copy number) level were selected. By fixing the copy number of these segments at a specified level, one can solve for the genome-wide bias of log ratio values as follows, and thus identify the genome-wide integer copy number profile.

$$R = {\rm{lo}}{{\rm{g}}_2}\left( {\frac{{\alpha \,{\rm{C}}{{\rm{N}}_T} + \left( {1 - \alpha } \right){\rm{C}}{{\rm{N}}_N}}}{2}} \right) - \delta $$

In the equation above, R represents the observed log ratio of read counts, *α* is the purity of the tumor sample, ${\rm{C}}{{\rm{N}}_T}$ and ${\rm{C}}{{\rm{N}}_N}$ are the integer copy number of tumor and normal samples at a locus, and *δ* is the genome-wide bias term. Given the value of tumor purity and copy number, *δ* is the only unknown in the equation. To favor solutions with less complex genomes, the copy number of regions with complete allelic balance was initially set to 2. If the resulting solution was deemed implausible (e.g., by implying chromosome or chromosome arm scale homozygous deletions), the copy number of regions with complete allelic balance was assigned to 4 and an alternative solution was found (Supplementary Fig. 10).

Details of the genomic segments selected to solve for the genome-wide bias term *δ* are as follows. In CGOV62, chromosomes 4 and 12 did not have allelic imbalance in any tumor samples. The solution assigning copy number two to these regions implied homozygous deletion of the p-arm of chrX in multiple samples; therefore, the simplest plausible solution assigned them to four copies. In CGOV63, chromosomes 6 and 15 did not have allelic imbalance in any of the tumor samples, and were assigned to two copies. No complete chromosome with absence of allelic imbalance across all tumor samples could be found in CGOV278. Therefore, four genomic regions with no allelic imbalance were selected for the normalization process above. These regions were chr8:38–69 Mb, chr12:62–85 Mb, chr18:7–19 Mb, chr20:23–35 Mb. The solution assigning these regions to two copies resulted in an implausible assignment of homozygous deletion to chr5:50–136 Mb. Therefore, assignment of four copies to the selected regions results in the simplest solution. In CGOV279, two genomic regions were selected for the normalization procedure: chr5: 64–131 Mb, chr20:17–36 Mb. Evaluation of log ratio values suggested that the two regions are present at different copy levels, as evidenced by a difference of ~0.60 in the log ratio values. The region on chr5, which had the lower log ratio level, was assigned to copy number 2. In CGOV280, chr16q had no allelic imbalance in any samples excluding the left FT STIC. Examination of log ratio values of chr16q in the left FT STIC supports a copy loss in that sample. The genome-wide bias term *δ* was determined by assignment of two copies to chr16q in the four samples with no allelic imbalance, and one copy in the left FT STIC.

### Subclonal hierarchy analysis

The tumor subclonality phylogenetic reconstruction algorithm SCHISM^25^ was used to infer tumor subclonal hierarchies from the set of confidently called somatic mutations in each patient. Given the estimates of genome-wide copy number profile, most copy number aberrations seem to occur early in the evolution of disease and are common across the lesions analyzed from each patient. Thus, the majority of somatic mutations can be assumed to occur following the acquisition of copy number aberrations, and can be present in cancer cells with multiplicity of one (one mutated copy per cell). Using this assumption, we can estimate mutation cellularity (or cancer cell fraction) from the observed reference and alternate read counts, and estimates of copy number, and tumor purity as follows.

$${V_{{\rm{exp}}}} = \frac{{m\alpha C}}{{\alpha \,{\rm{C}}{{\rm{N}}_T} + \left( {1 - \alpha } \right){\rm{C}}{{\rm{N}}_N}}}$$

In the equation above, ${V_{\rm{exp}}}$ is the expected variant allele frequency of the mutation, *m* is the multiplicity of the mutation which is set to 1, *α* is the purity of the tumor sample, *C* is the cellularity of the mutation, and ${\rm{C}}{{\rm{N}}_T}$ and ${\rm{C}}{{\rm{N}}_N}$ are the integer copy number of tumor and normal sample at the locus of the mutation. The observed alternate read count of the mutation can be modeled as a binomial random variable drawn from a distribution with probability parameter equal to ${V_{{\rm{exp}}}}$ and number of trials equal to the total sequence coverage of the mutation. We calculated the likelihood for observation of the alternate read counts for cellularity values spanning the range of 0–1 in increments of 0.01, and derived the maximum likelihood estimate and confidence interval for the mutation cellularity.

To obtain reliable estimates of mutation cellularity, we clustered mutations by joint presence or absence across the available tumor samples. This approach makes phylogenetic reconstruction more tractable and the cellularity of the resulting clusters can be estimated with higher accuracy than that of individual mutations. For each patient, a mutation was called as present or absent in each of the available tumor samples (10 samples from CGOV62, 6 samples from CGOV63, 5 samples from CGOV280, 4 samples from CGOV279, and 3 samples from CGOV278). To call the mutation present, we used a minimum allele frequency of 2% and 2 distinct mutant reads. Mutation clustering was performed by a greedy algorithm. Tumor purity in each tumor sample was estimated as the read count fraction of *TP53* mutation in each patient. Each patient harbored a single distinct *TP53* mutation that was present in all tumor samples, and we assumed the wild-type allele was lost, as supported by the ubiquitous LOH of chromosome 17. To derive a more comprehensive view of the evolution of these samples, we extended the original SCHISM framework to model acquisition of large scale somatic copy number alterations, which can be detected by analysis of allelic imbalance (including LOH). First, we extracted a set of high confidence genomic regions with ubiquitous, partially shared, or private LOH in tumor samples of each patient (Methods section). These regions of LOH served as binary features that could be used for evolutionary analysis, and were clustered into LOH feature groups with identical patterns of presence or absence across samples (Fig. 2). Each LOH feature group was compared to the somatic mutation clusters in each patient, with respect to its pattern of presence or absence across samples. In cases where a mutation cluster with the identical pattern could be found, the cluster and the LOH feature group were assumed to have occurred together in the course of tumor evolution. Otherwise, the LOH feature groups were modeled as distinct features, and added in post-hoc analysis by application of the lineage precedence rule from SCHISM; which requires cellularity of ancestor alterations to be greater than or equal to cellularity of descendant alterations in all tumor samples.

SCHISM was run with the above inputs and default parameter settings to infer the order of somatic alterations and thus define subclonal hierarchy in each patient. SCHISM software is freely available for non-profit use at http://karchinlab.org/appSchism.

Evolutionary trees resulting from SCHISM analysis were compared with those derived by maximum parsimony phylogeny using PHYLIP (Phylip-3.695, PARS method). For CGOV280, an adjustment to the tree was applied to account for multiple subclones in Right FT STIC.

### Estimating an evolutionary timeline

Following the approach of Jones et al.^29^, the observed data are the number of somatic mutations in the STIC (${n_j}$), the number of mutations in the metastasis (${n_k}$), and the age at which the patient was diagnosed (${t_k}$), where somatic mutations include both sequence and structural alterations. Unknown is the birthdate (${t_j}$) of the cell that was the last common ancestor of the STIC and the metastasis. Assuming the mutation rate of somatic passenger mutations and the length of the cell cycle is constant, the number of somatic mutations in the metastasis cell that were present in the STIC follows a binomial distribution with parameters *n*_*k*_ and probability *t*_*j*_/*t*_*k*_. As *t*_*j*_ is unknown, we posit a conjugate beta probability distribution on the rate *t*_*j*_/*t*_*k*_ with shape parameters *a* and *b* estimated from previous studies as described below. The posterior distribution of *t*_*j*_/*t*_*k*_ is *β* (*a* + *n*_*j*_, *b* + *n*_*k*_−*n*_*j*_) from which 90% highest posterior density intervals can be constructed with point estimates for the birthdate reported as the posterior mean. For simplicity, we refer to the highest posterior density as a confidence interval. To construct a prior for *t*_*j*_/*t*_*k*_, we draw on a previous study of four colorectal cancer patients^29^ where a small number of additional passenger mutations were acquired by the cell that gave birth to the metastasis. On average, 95% of the mutations in the original adenocarcinoma were present in the metastases. We center the mean for the beta prior at 0.95 using shape parameters *a* = 34 and *b* = 1.6. Our prior is equivalent to one patient having 34 passenger somatic mutations in the original lesion and 1.6 additional mutations to be acquired by cells that gave birth to the metastases. For patients with three samples in a linear tree as determined by evolutionary analyses (say, samples *j, k*, and *l* where sample *j* is the STIC, *l* is the metastasis, and *k* is an intermediate sample), we first derived the posterior distribution for *t*_*k*_ comparing mutations in samples *k* and *l*. Next, we derived the posterior distribution of *t*_*j*_ integrating over all possible values of *t*_*k*_, thereby fully incorporating the uncertainty of the intermediate timepoint in the estimate of *t*_*j*_. We evaluated three additional prior models, and found that that posterior inference under these alternative models given by 90% credible intervals for *t*_*k*_−*t*_*j*_, results in qualitatively similar timelines among different lesions in tumor progression.

### Data availability

Sequence data have been deposited at the European Genome-phenome Archive, which is hosted at the European Bioinformatics Institute, under study accession EGAS00001002589.

## Electronic supplementary material

Supplementary Information

Supplementary Data 1

Supplementary Data 2

Supplementary Data 3

Supplementary Data 4

Supplementary Data 5

Supplementary Data 6

Supplementary Data 7

Supplementary Data 8

Supplementary Data 9

Supplementary Data 10

Supplementary Data 11
