# Supplementary material for: High grade serous ovarian carcinomas originate in the fallopian tube
Source: Nat Commun. 2017 Oct 23;8:1093. doi: 10.1038/s41467-017-00962-1 (PMC5653668; doi:10.1038/s41467-017-00962-1)
Supplement: Supplementary file 1 — Supplementary Information [file 41467_2017_962_MOESM1_ESM.pdf]

Description of Supplementary Files

File Name: Supplementary Information
Description: Supplementary Figures

File Name: Supplementary Data 1
Description: Summary of Patient and Sample Characteristics

File Name: Supplementary Data 2
Description: Summary of Next-Generation Sequencing Analyses

File Name: Supplementary Data 3
Description: Targeted Sequencing Panel

File Name: Supplementary Data 4
Description: Somatic Sequence Alterations

File Name: Supplementary Data 5
Description: Somatic Sequence Alterations used for Evolutionary Cluster Analyses

File Name: Supplementary Data 6
Description: Recurrent Somatic Sequence Alterations

File Name: Supplementary Data 7
Description: Regions of Allelic Imbalance in CGOV62

File Name: Supplementary Data 8
Description: Regions of Allelic Imbalance in CGOV63

File Name: Supplementary Data 9
Description: Regions of Allelic Imbalance in CGOV280

File Name: Supplementary Data 10
Description: Regions of Allelic Imbalance in CGOV279

File Name: Supplementary Data 11
Description: Regions of Allelic Imbalance in CGOV278

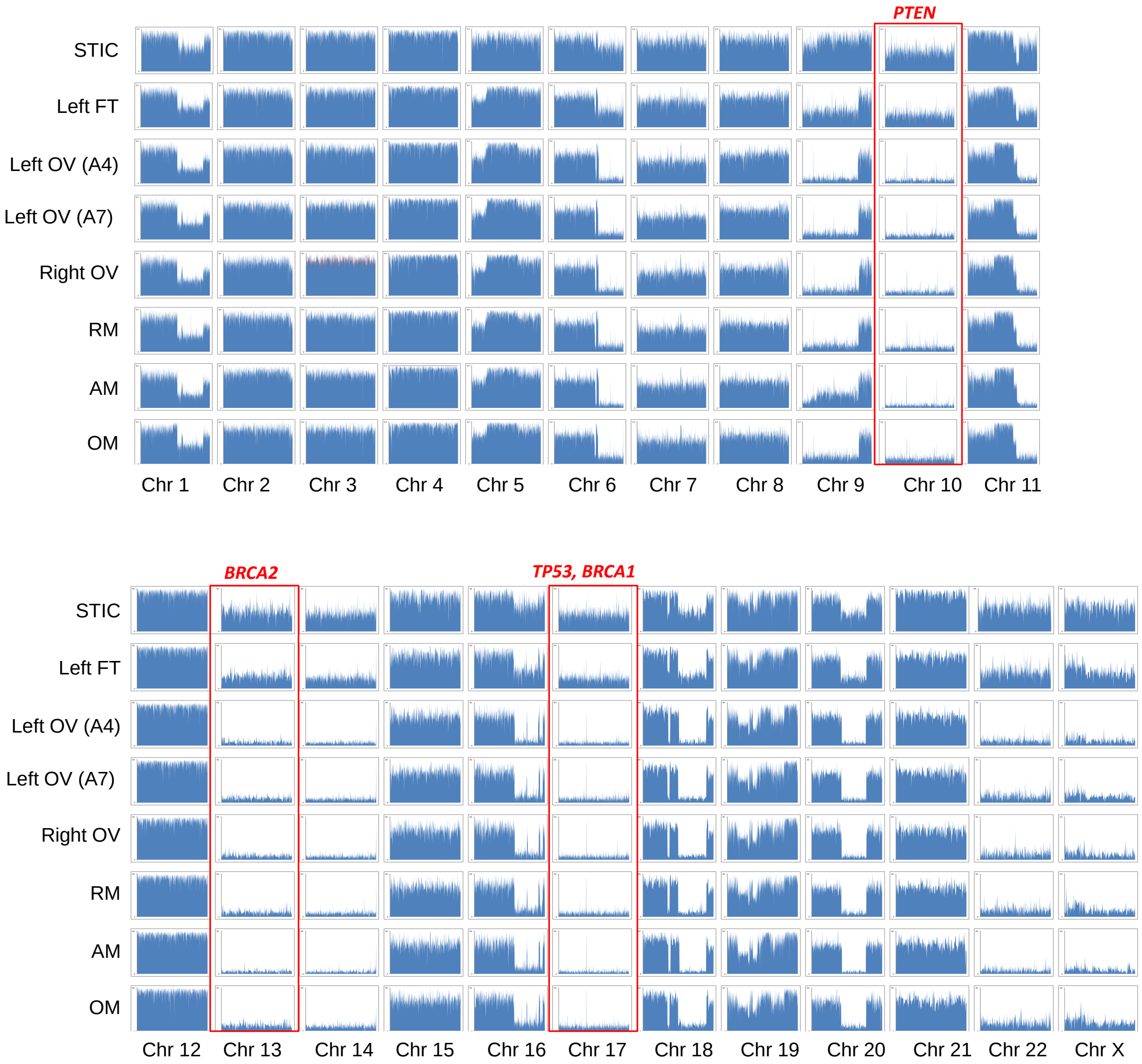

Supplementary Figure 1. Loss of heterozygosity analyses for CGOV62. The graphs represent B allele frequencies (BAFs) for the indicated chromosomes. A value of 0.5 indicates a heterozygous genotype (AB) whereas allelic imbalances in tumor samples are observed as a deviation from 0.5. BAF values of 0 typically indicate loss of heterozygosity, although normal contaminating tissue may limit the minimum observed value. Graphs for CGOV62 include left fallopian tube STIC, left fallopian tube tumor, left ovarian tumor block A4, left ovarian tumor block A7, right ovarian tumor, rectal metastasis, appendiceal metastasis, and omental metastasis. LOH events that contain *PTEN*, *BRCA2*, *TP53* and *BRCA1* genes are indicated by the red boxes.

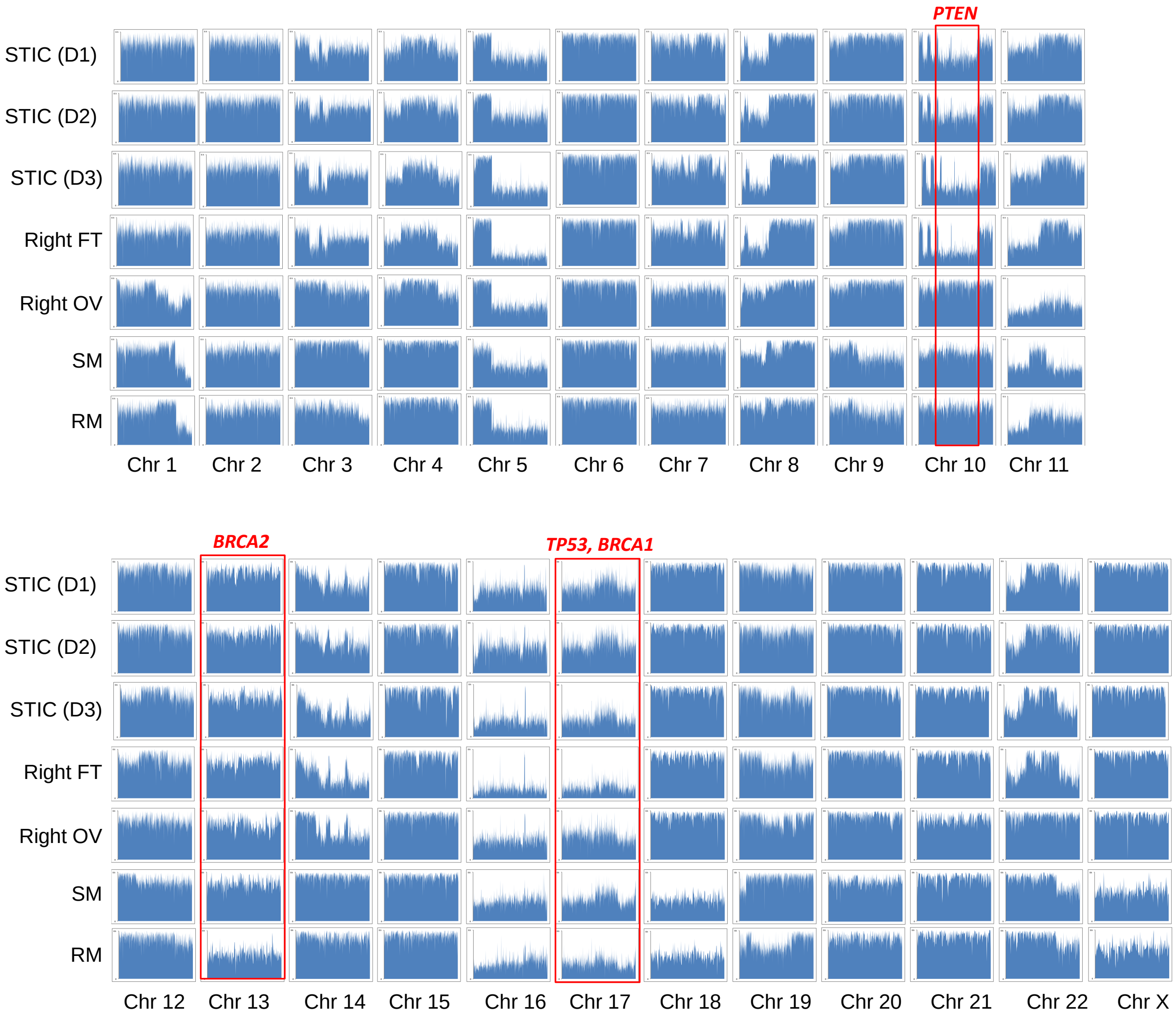

Supplementary Figure 2. Loss of heterozygosity analyses for CGOV63. The graphs represent B allele frequencies (BAFs) for the indicated chromosomes. A value of 0.5 indicates a heterozygous genotype (AB) whereas allelic imbalances in tumor samples are observed as a deviation from 0.5. BAF values of 0 typically indicate loss of heterozygosity, although normal contaminating tissue may limit the minimum observed value. Graphs for CGOV63 include STIC block D1, STIC block D2, STIC block D3 , right fallopian tumor, right ovarian tumor, sigmoid metastasis, rectal metastasis. LOH events that contain *PTEN*, *BRCA2*, *TP53* and *BRCA1* genes are indicated by the red boxes.

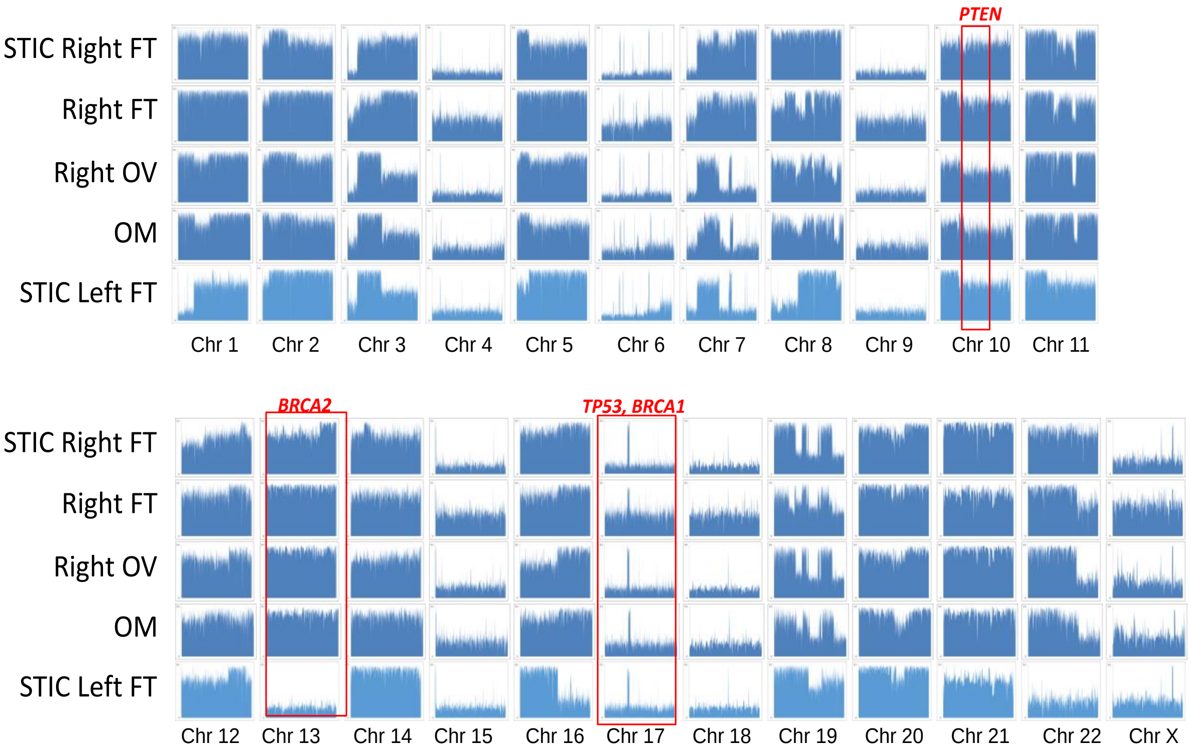

Supplementary Figure 3. Loss of heterozygosity analyses for CGOV280. The graphs represent B allele frequencies (BAFs) for the indicated chromosomes. A value of 0.5 indicates a heterozygous genotype (AB) whereas allelic imbalances in tumor samples are observed as a deviation from 0.5. BAF values of 0 typically indicate loss of heterozygosity, although normal contaminating tissue may limit the minimum observed value. Graphs for CGOV280 include STIC of the right fallopian tube, right fallopian tube tumor, right ovarian tumor, omental metastasis, stic of the left fallopian tube. LOH events that contain *PTEN*, BRCA2, *TP53* and *BRCA1* genes are indicated by the red boxes.

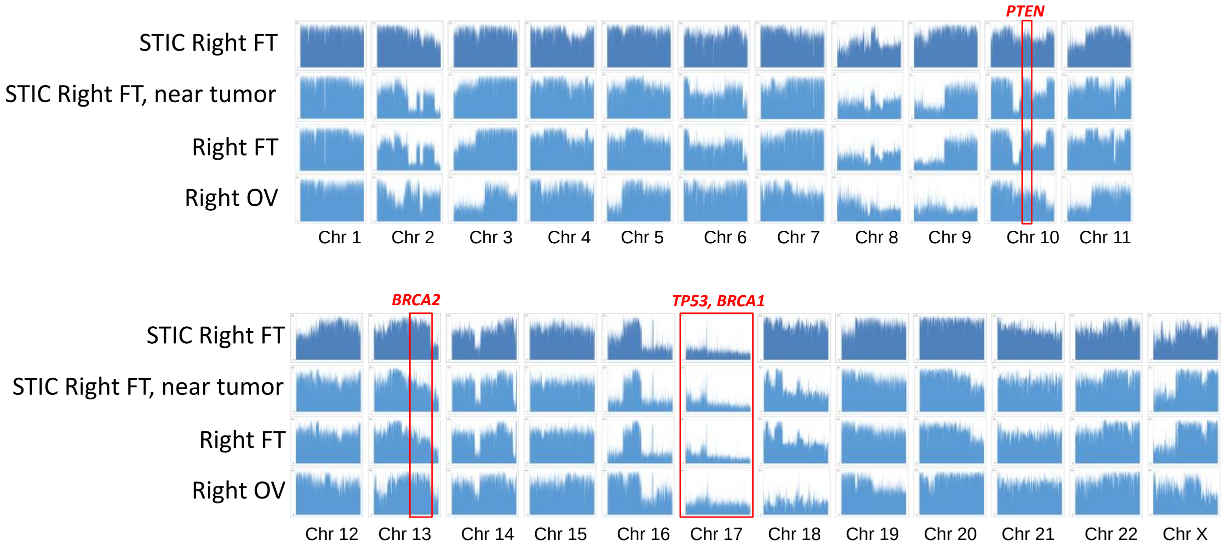

Supplementary Figure 4. Loss of heterozygosity analyses for CGOV279. The graphs represent B allele frequencies (BAFs) for the indicated chromosomes. A value of 0.5 indicates a heterozygous genotype (AB) whereas allelic imbalances in tumor samples are observed as a deviation from 0.5. BAF values of 0 typically indicate loss of heterozygosity, although normal contaminating tissue may limit the minimum observed value. Graphs for CGOV279 include STIC of the right fallopian tube, STIC of the right fallopian tube close to the tumor, right fallopian tube tumor, right ovarian tumor. LOH events that contain *BRCA2*, *TP53* and *BRCA1* genes are indicated by the red boxes, while the LOH region in chromosome 10 of this sample does not overlap with the *PTEN* gene.

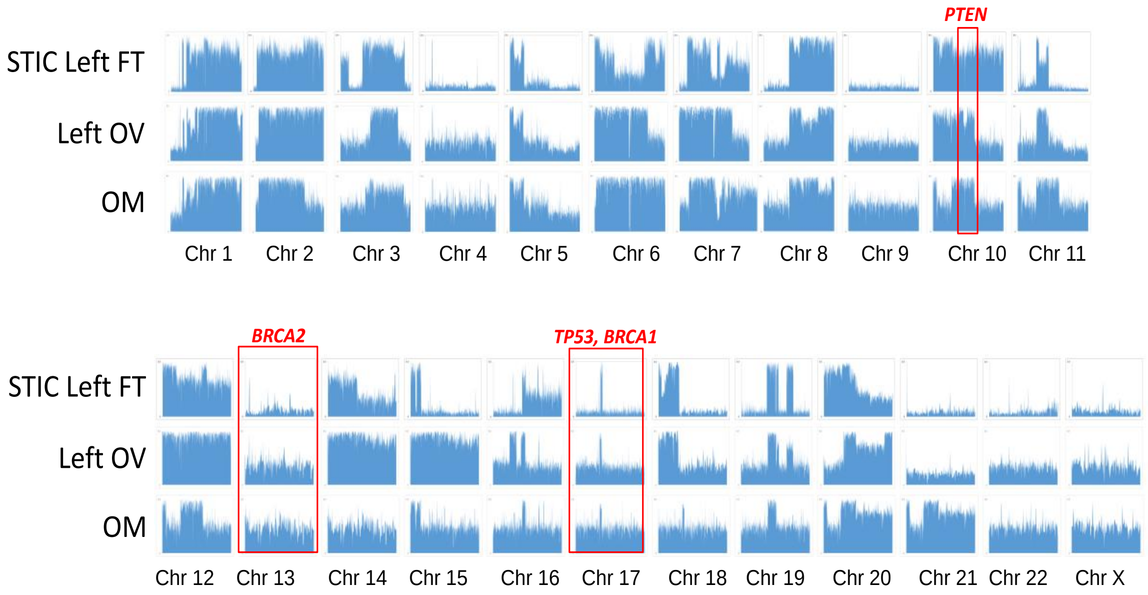

Supplementary Figure 5. Loss of heterozygosity analyses for CGOV278. The graphs represent B allele frequencies (BAFs) for the indicated chromosomes. A value of 0.5 indicates a heterozygous genotype (AB) whereas allelic imbalances in tumor samples are observed as a deviation from 0.5. BAF values of 0 typically indicate loss of heterozygosity, although normal contaminating tissue may limit the minimum observed value. Graphs for CGOV278 include STIC of left fallopian tumor, left ovarian tumor, omental metastasis. LOH events that contain *BRACA2*, *TP53* and *BRCA1* genes are indicated by the red boxes, while the LOH region in chromosome 10 of this sample does not overlap with the *PTEN* gene.

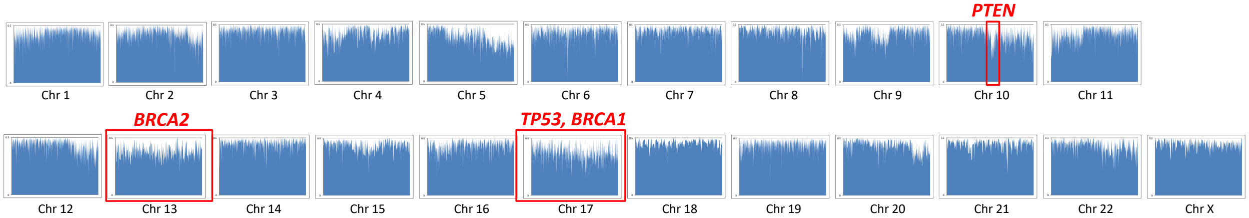

Supplementary Figure 6. Loss of heterozygosity analyses for CGOV65. The graphs represent B allele frequencies (BAFs) for the indicated chromosomes for the STIC lesion. A value of 0.5 indicates a heterozygous genotype (AB) whereas allelic imbalances in tumor samples are observed as a deviation from 0.5. BAF values of 0 typically indicate loss of heterozygosity, although normal contaminating tissue may limit the minimum observed value. LOH events that contain *PTEN*, *BRACA2*, *TP53* and *BRCA1* genes are indicated by the red boxes.

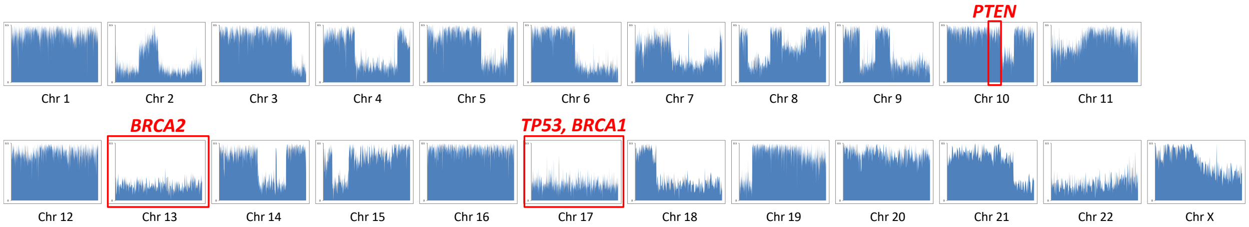

Supplementary Figure 7. Loss of heterozygosity analyses for CGOV64. The graphs represent B allele frequencies (BAFs) for the indicated chromosomes for the STIC lesion. A value of 0.5 indicates a heterozygous genotype (AB) whereas allelic imbalances in tumor samples are observed as a deviation from 0.5. BAF values of 0 typically indicate loss of heterozygosity, although normal contaminating tissue may limit the minimum observed value. LOH events that contain *BRACA2*, *TP53* and *BRCA1* genes are indicated by the red boxes, while the LOH region in chromosome 10 of this sample does not overlap with the *PTEN* gene.

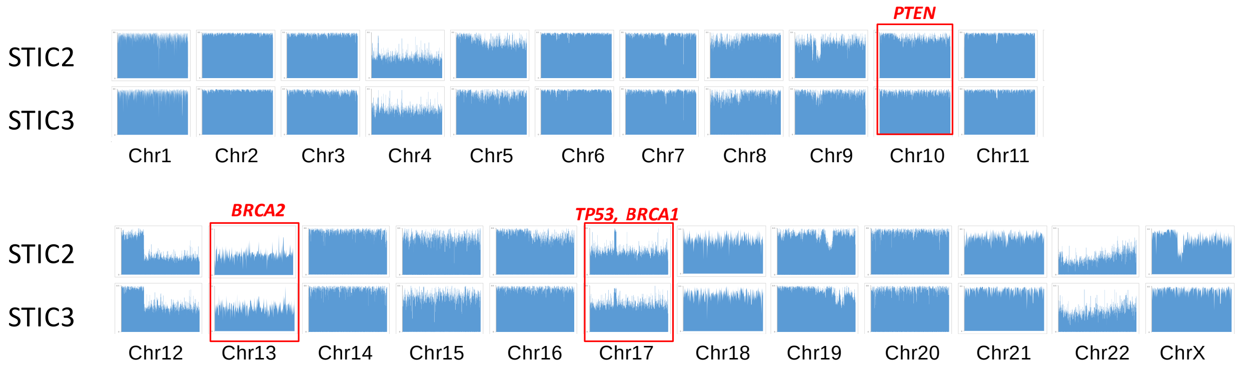

Supplementary Figure 8. Loss of heterozygosity analyses for CGOV303. The graphs represent B allele frequencies (BAFs) for the indicated chromosomes for the STIC lesion. A value of 0.5 indicates a heterozygous genotype (AB) whereas allelic imbalances in tumor samples are observed as a deviation from 0.5. BAF values of 0 typically indicate loss of heterozygosity, although normal contaminating tissue may limit the minimum observed value. LOH events that contain *BRACA2*, *TP53* and *BRCA1* genes are indicated by the red boxes, while no LOH in chromosome 10 containing *PTEN* was observed.

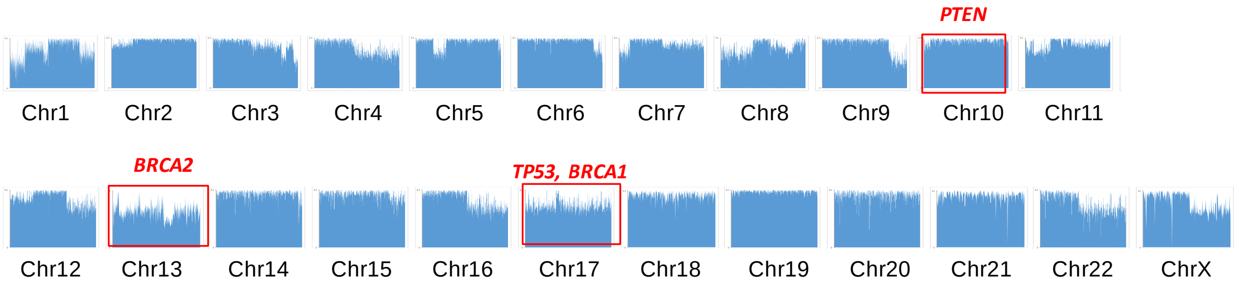

Supplementary Figure 9. Loss of heterozygosity analyses for CGOV304. The graphs represent B allele frequencies (BAFs) for the indicated chromosomes for the STIC lesion. A value of 0.5 indicates a heterozygous genotype (AB) whereas allelic imbalances in tumor samples are observed as a deviation from 0.5. BAF values of 0 typically indicate loss of heterozygosity, although normal contaminating tissue may limit the minimum observed value. LOH events that contain *BRACA2*, *TP53* and *BRCA1* genes are indicated by the red boxes, while no LOH in chromosome 10 containing *PTEN* was observed.

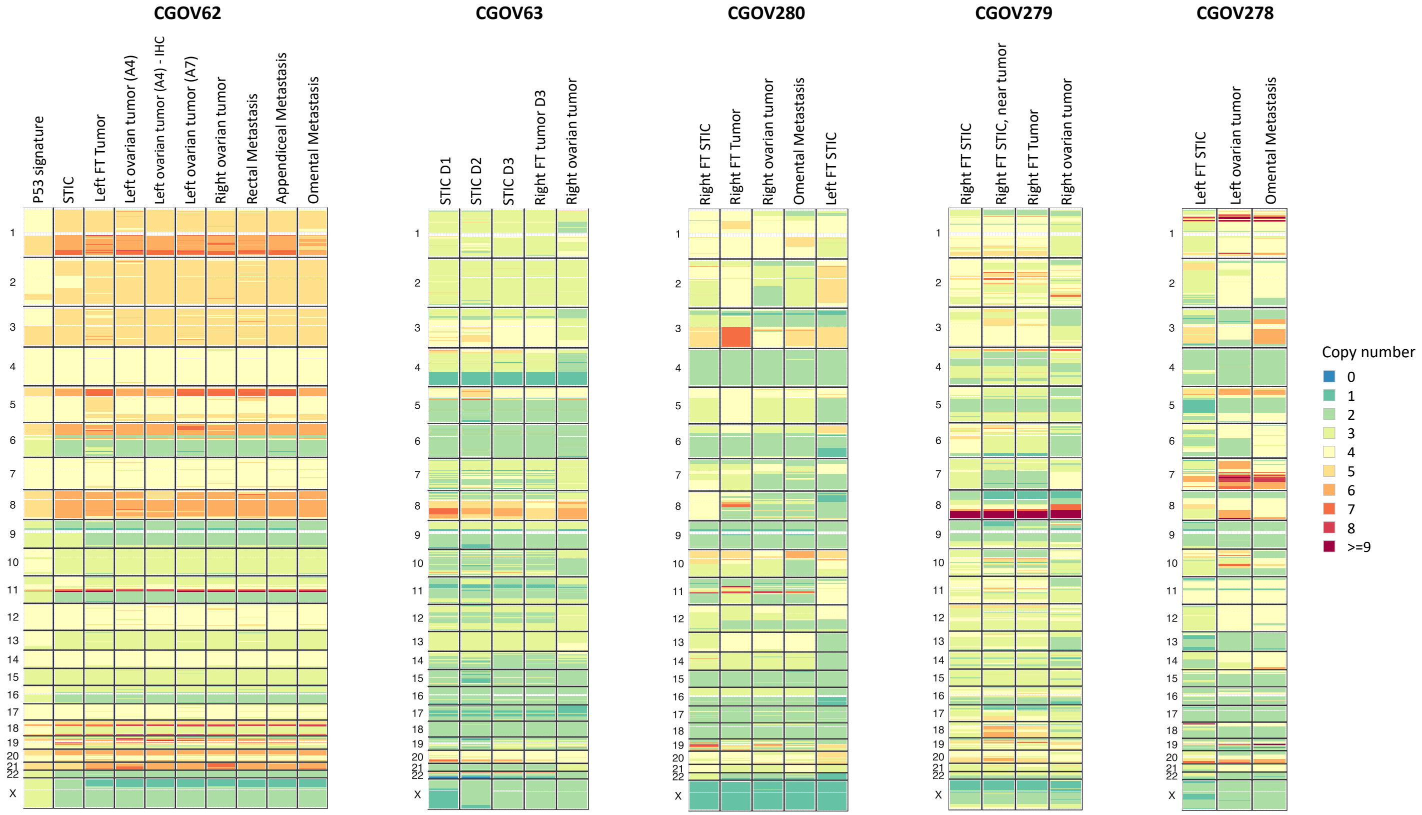

Supplementary Figure 10. Genome-wide copy number profile. Copy number ratio of genomic bins were determined based on target and off-target reads from whole exome sequencing. The raw copy ratio were corrected for GC-content, repetitive sequences, and target capture enrichment using CNVKit, and then converted to integer copy levels by accounting for tumor purity and average genome-wide ploidy (Methods, copy number analysis). The resulting copy number profiles suggest a relatively consistent pattern of copy number aberrations across the lesions analyzed from each patient.

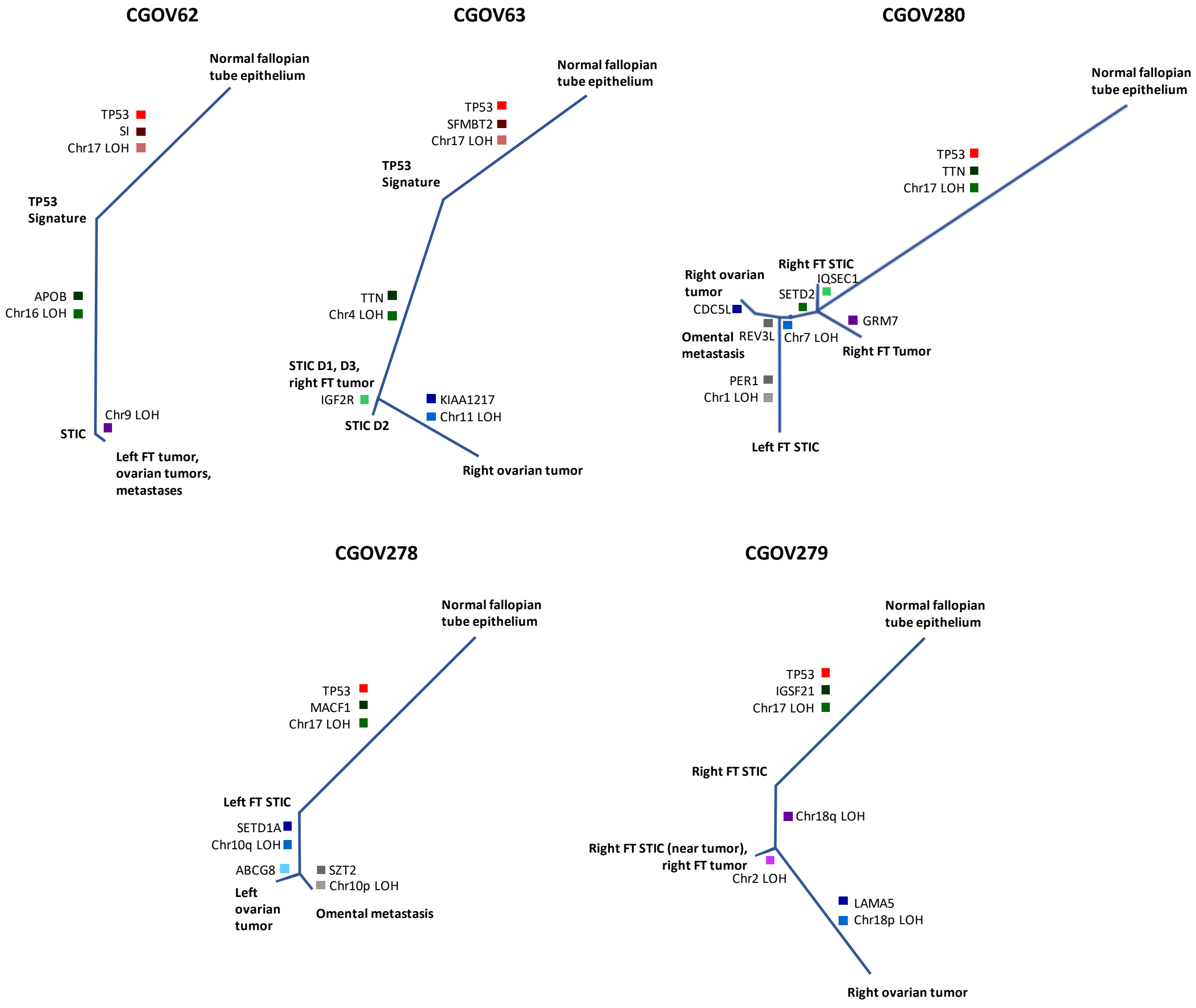

Supplementary Figure 11. Schematic of tumor evolution using PHYLIP. Visualization of evolutionary trees from each patient based on mutational and LOH changes. Branch lengths are proportional to the number of somatic alterations.
